# Two Cases of Symptomatic Tailgut Cysts

**DOI:** 10.3390/jcm13175136

**Published:** 2024-08-29

**Authors:** Jan Wojciechowski, Tomasz Skołozdrzy, Piotr Wojtasik, Maciej Romanowski

**Affiliations:** Department of General and Oncological Surgery, Pomeranian Medical University, 70-204 Szczecin, Poland

**Keywords:** tailgut cyst, rectorectal cystic, malignancy risk

## Abstract

Tailgut cysts are rare lesions which are found in the rectorectal space. They develop in the final section of the intestine from which the rectum and anus extend and vary from being asymptomatic to symptomatic due to pressure on organs or nerves. Tailgut cysts are more common in females, usually between 30 and 60 years of age. They are thought to be benign, with variable malignancy risks. Surgical excision followed by histological examination is the gold standard of treatment, but access and approach to tailgut cysts depend on the location and morphology of the lesion. We present two symptomatic cases of this very rare pathology. In both cases, the cyst and coccyx bone were successfully excised using different approaches. The first patient was a 40-year-old woman with a large cyst which caused morning tenesmus, urinary outflow disorders and painful ovulation. Due to the cyst size, laparotomy was performed, and a combined approach was used. The second patient is a 36-year-old woman with co-existing endometriosis and a cyst causing pain in the sacral spine, constipation and tenesmus. The tumor was excised using a Kraske approach, and due to the infiltration of the coccyx bone it was removed using an osteotome. In this patient, perforation of the cyst was also observed. Both patients completed follow-ups involving regular surgical check-ups and MRI scans. Descriptions of different symptoms and surgical approaches make our study an important source of knowledge for diagnosing and treating these very rare tumors.

## 1. Introduction

The presacral space is a potential space that becomes a true space when a mass grows within it [1]. The anterior boundary of the space is formed by the mesorectum, and the posterior boundary is formed by the anterior aspect of the sacrum. Inferiorly, it extends up to the rectosacral fascia, which passes forward from the S4 vertebrae to the rectum ~3 to 5 cm proximal to the anorectal junction. Superiorly, the space extends up to the peritoneal reflection. The ureters, the sacral nerve roots, the lateral stalks of the rectum and the iliac vessels bound the lateral extent of the presacral space. The presacral space contains loose connective tissue, the middle sacral artery, the superior rectal vessels and branches of the sympathetic and parasympathetic nervous systems [2].

Differential diagnoses of tumors in this space include lesions such as fistulas, abscesses, granulomas, osteomas, osteosarcomas, leiomyomas, fibrosarcomas, neurofibromas and ganglioneuromas, with tailgut cysts comprising only a small fraction of retrorectal tumors that can arise in this potential space [3,4].

Presacral tumors are divided into congenital, neurogenic, inflammatory and miscellaneous tumors. Congenital tumors are the most common tumors of the presacral space, accounting for 55% to 70% of all lesions [2]. They are further divided into developmental cysts, sacrococcygeal chondromas and anterior meningoceles. A total of 60% of congenital presacral cysts are developmental cysts, whose characteristic feature is that they can arise from any of the three embryonic germ layers [2]. Developmental cysts are further divided into epidermoid cysts, dermoid cysts, enterogenous cysts, teratomas and finally tailgut cysts. Contrary to epidermoid and dermoid cysts, which contain only squamous epithelium, tailgut cysts can have columnar and transitional epithelium. In contrast to duplication cysts, they do not have organized smooth muscle fibers within the cyst wall and do not contain neural plexuses. Tailgut cysts are described as multilocular and multicystic. They may be lined by different kinds of epithelia like stratified squamous, mucinous, ciliated columnar, stratified columnar or transitional. Different kinds of epithelia may even appear within the same cyst. A characteristic feature of tailgut cysts which differentiates them from other presacral tumors is the origin of the cells, which are primitive gut remnants [2].

Tailgut cysts develop in the final section of the intestine from which the rectum and anus extend. The embryo develops a tail on approximately the fifth week of gestation that regresses during the eighth week of fetal life. The ectoderm then invaginates to the end of the gut and the anus forms. However, if this process is disturbed, a stray fragment of hindgut remains, and the tailgut cyst is formed [1].

Tailgut cysts are more common in females, with a 5:1 ratio of predominance [3]; furthermore, a study by Mathis et al. showed an even larger female dominance, as out of 31 tailgut cyst cases between 1985 and 2008, 28 of patients were women, which raises the ratio to 9:1. Tailgut cysts usually appear between the ages of 30 and 60, but can be present at any age [5,6,7]. This differentiates them from presacral teratomas, which are most commonly seen in infancy, and are rare beyond the second decade of life [2].

Because of these lesions’ rarity and their potential risk of malignancy, we present two cases, which occurred in our clinic, to increase awareness of this type of lesion.

## 2. Cases Presentation

**Figure 1:**
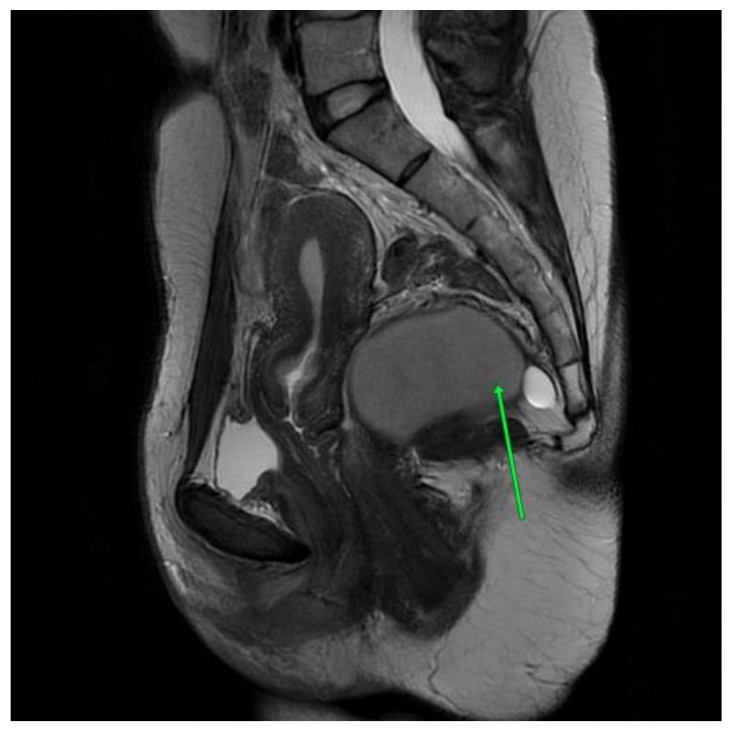
First patient MRI scan of the lesser pelvis. Green arrow shows the lesion.

I First patient:

The first patient is a 40-year-old woman with large intestinal hyperplastic polyps and hemorrhoids. The pelvis minor tumor was diagnosed in 2016 during a routine ultrasound, and the patient underwent an unsuccessful surgery the same year. The tumor was asymptomatic, but in 2021, the patient started to experience morning tenesmus, urinary outflow disorders and painful ovulation. The physical examination showed left-sided thickness in a per vaginam examination. An MRI scan the with contrast of the lesser pelvis revealed a cystic, multilocular mass measuring 8.9 cm × 8.1 cm× 6.3 cm. This lesion shifted the rectum forward and to the left. It pressured the reproductive organ. The MRI scan aroused suspicion of a tailgut cyst [Figure 1].

Due to the symptomatic character of the tumor, a laparotomy was performed. The peritoneum was opened with a midline incision. Aside from an inflamed left uterine appendage, no pathological changes were found. Then, the peritoneum was opened on the right side, from the rectum. A large cystic tumor was visible, filling presacral and precoccygeal areas. The tumor was dissected from the mesorectum of the sidewall of the pelvis, from the reproductive organ from below and from the coccyx bone going down to the pelvic floor where infiltration of the coccyx was visible. The patient was shifted onto their stomach and another midline incision was performed, which made the removal of the tumor and coccyx bone possible. Also, two accidentally detected small cystic tumors in the external anal sphincter area were removed. After checking tightness of the rectum with a catheter syringe and placing a Redon drain in the presacral area, the wound of the sacrococcygeal area was sutured. The patient was shifted back onto their back and the abdominal integuments were closed.

Due to the extent of the procedure, the patient was transported to the Intensive Care Unit on schedule. Ischemia of the left lower limb was suspected, and a Doppler ultrasound was performed which showed no blood circulation disorders. At 24 h after the operation, the fully awake patient was transported in good general condition to the Surgery Clinic. Clinical nutrition was implemented, and empirical antibiotic therapy was prolonged. In the following days the patient regained full mobility and resumed eating a complete diet. Postoperative wounds were healing properly. The patient was discharged from the hospital 8 days after the procedure in good physical condition.

**Figure 2:**
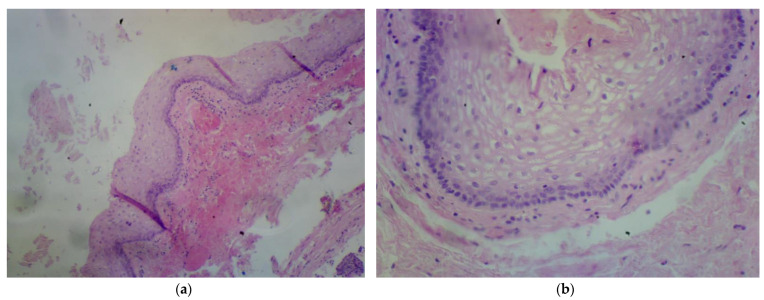
First patient histopathological examination. (**a**) Tissue at 4× magnification. (**b**) Tissue detail at 10× magnification.

In the histopathological examination, a multilocular, cystic tumor was described. In the wall, fibrous tissue and smooth muscle tissue were visible. Cystic spaces were padded with cuboidal epithelium and stratified squamous epithelium without signs of cell atypia. Malignant transformation was excluded and the tailgut cyst diagnosis was favored [Figure 2].

The patient remains under the control of the Surgery Clinic, with a check-up every six months. At the first check-up, she did not report any symptoms. The patient will undergo control imaging once a year.

II Second Patient

**Figure 3:**
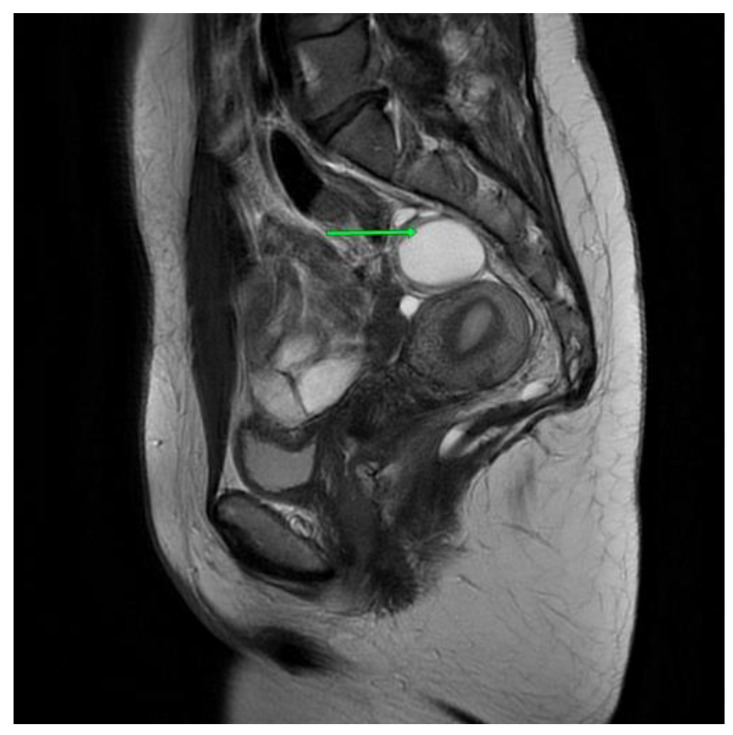
Second patient MRI scan of lesser pelvis. Green arrow shows the cyst.

The second patient is a 36-year-old woman with a tumor of the lesser pelvis and endometriosis. In 2022, the patient started to experience pain in the sacral spine, which increased when she was sitting and after physical activity. The patient also complained about constipation and tenesmus. An ultrasound showed a tumor in the lesser pelvis. An MRI scan showed a cystic, multilocular tumor measuring 3.3 cm × 3.3 cm × 3.6 cm [Figure 3]. The signal had high intensity in the T1-weighted phase. The cyst pressured the rectum. The patient underwent unsuccessful laparoscopy and laparotomy in the Gynecology and Obstetrics Clinic.

**Figure 4:**
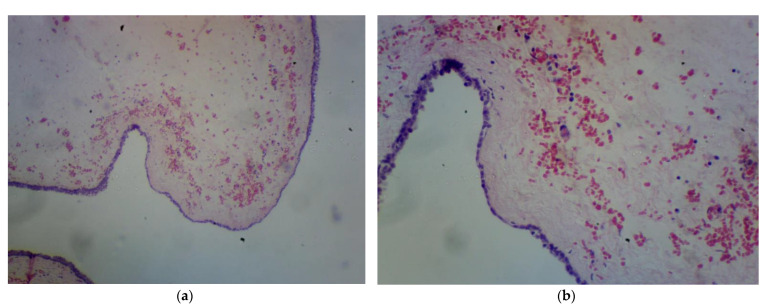
Second patient histopathological examination. (**a**) Tissue at 4× magnification. (**b**) Tissue detail at 10× magnification.

In the Surgery Clinic, the tumor and the coccyx bone were excised following the Kraske approach. A midline incision was made between coccyx bone and the anus and extended in the midline supracoccygeal and suprasacral areas. Then, a coccyx bone sample was excised using an osteotome. A precoccygeal cystic tumor in the retrorectal space was visible. The tumor was dissected from the walls of the rectum and mesorectum fatty tissue, which was difficult due to the blurred boundaries of the tumor. During dissection, the tumor perforated, and greenish, thick and odorless fluid started to secrete. The fluid was taken for culture. The tumor was then dissected from the rectum, external anal sphincter and levator ani. The tumor was completely excised, keeping the external anal sphincter intact. A Redon drain was placed, and the wound was sutured. Histological examination showed a benign tailgut cyst [Figure 4].

After the procedure, the patient was transported to the Surgery Clinic. She was treated with empirical antibiotic therapy and fluid therapy. The culture was negative. The wound healed properly, and the patient was discharged from the hospital 9 days after the procedure in good physical condition.

The patient remained under control of the Surgery Clinic with a check-up every six months and an MRI scan every year. The first check-up showed the wound to have healed. However, the patient reported periodic stinging around the anus. In a rectal examination, no pathologies were visible. A colonoscopy was ordered, which confirmed this conclusion. The patient did not report these complaints in the next check-up.

**Figure 5:**
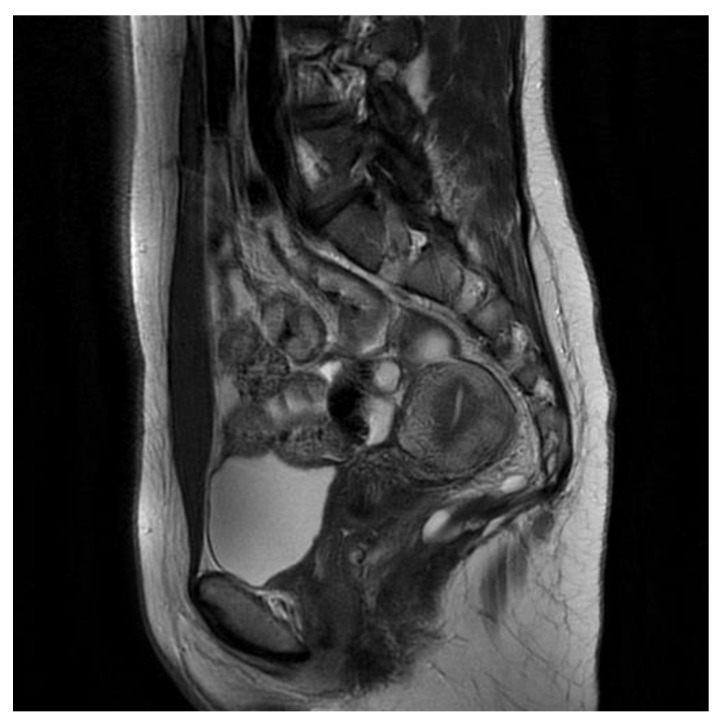
Second patient MRI scan of the lesser pelvis eight months after the procedure.

In the first MRI scan, eight months after the procedure, a small amount of fluid was visible in the operated area. Some small cystic, thin-walled structures were also visible, and will be monitored in future imaging [Figure 5].

## 3. Discussion

Tailgut cyst presentation varies from asymptomatic incidental findings to symptomatic tumors due to pressure on organs and nerves. In a study by Mathis et al. [4], out of 31 patients, in 14 of them the cyst was discovered incidentally, while in other cases it presented with symptoms.

The patients’ symptoms include lower back or perineal pain, constipation, urinary or fecal incontinence, urinary retention, rectal bleeding and sexual or neurological dysfunctions. A history of recurrent anal sinus, fistulae or abscesses is also an alarming sign [2]. In the case of our patients, both tailgut cysts were symptomatic. The first patient experienced morning tenesmus, urinary outflow disorders and painful ovulation, and the second patient suffered from pain in the sacral spine, which increased while she was sitting and after physical activity. She also complained about constipation and tenesmus. If infected, tailgut cysts can present with signs and symptoms that are similar to perianal or pelvic abscesses [5]. Most of the time, the symptoms are triggered due to the cyst size and the pressure that it causes. In a research study by Mathis et al. [4], the median cyst diameter was 4.4 cm. In our first patient, the tumor was twice as big, and in the second patient, it was slightly smaller.

In our second case, the tailgut cyst was diagnosed in a patient with co-existing endometriosis, which to the best of our knowledge is the first reported case of these two diseases coinciding. In the literature, two cases were reported where suspected endometriosis was in fact a tailgut cyst [8,9]. Endometriosis can present with similar symptoms, and a tailgut cyst can be mistaken for a chocolate cyst [10,11]. Due to endometriosis being a common disease in women, the risk of misdiagnosis of a tailgut cyst is significant.

Tailgut cysts are usually benign tumors, however malignant transformation is possible and was reported in various research studies. For years the risk of malignancy was thought to be quite low, estimated to be 2%; however, in the research study of Mathis et al. [4], malignant transformation was reported in 4 cases, which makes the ratio increase to 14%. Three of these cases were adenocarcinomas, and one was a carcinoid [4]. Malignant tailgut cysts can be positive for CEA, as the majority of colorectal cancers are, due to a dysplasia–carcinoma sequence related to p53 gene mutation [12]. A total of 80% of the reported malignant cases are adenocarcinomas and neuroendocrine tumors [1]. Individual cases include sarcoma and transitional cell carcinoma [13]. Research from 2018 by Kaistha et al. [14] reported twenty-seven cases of malignant transformation of the tailgut cyst. Adenocarcinoma has shown a high recurrence rate within 5 months to 3 years. Recurrence ratio of the benign tailgut cysts is low; Mathis et al. [4] reported only one case of it. Due to the risk involved, complete excision of the cyst is needed, and the patient needs to be followed up with. For supervision, an annual digital rectal exam and CT scan in postoperative one year and five years after the operation are recommended [4].

Patient follow-up is also important in order to prevent recurrence and correctly diagnose late complications. In our patients’ cases, we decided to conduct check-ups with digital rectal exams every six months, and an MRI scan yearly. We decided to follow this strict approach as late complications after tailgut cyst excision were reported in the literature, such as intersphinteric anal fistula reported in research by Volk et al. [15]. In our second patient’s case, at the first check-up she complained about periodic stinging around the anus. In a short time, a colonoscopy was performed in order to check for possible perforations or fistulas.

CT and MRI are the best imaging modalities for diagnosis, often differentiating between benign and malignant pathology. MRI has a sensitivity of 90% and a specificity of 98%, allowing for the assessment of tumor location, size, morphology and interface [16]. The risk of malignancy appears when in CT or MRI imaging the wall of a cyst is irregular or thickened. MRI can precisely image soft tissue and evaluate the presence of bony invasion or nerve involvement [14]. In the cases of our patients, both had an MRI scan performed.

Carrying out preoperative percutaneous biopsy is disputed. Mathis et al. [4] recommends a percutaneous preoperative presacral biopsy if the malignancy possibility is high [2]; however, for malignant lesions, there is significant risk of seeding. Preoperative biopsy as an invasive examination involves some risk, such as infection or hematoma, and may increase recurrence rates [16]. The development of imaging techniques makes preoperative biopsies unnecessary in most cases, especially if the tumor is considered to be resectable. In the cases of our patients, no preoperational biopsies were performed.

Access and approach to a tailgut cyst depend on the location and morphology of it. The most common surgical approach described for tailgut cyst excision is via a posterior parasacral incision, with the goal of the complete removal of the cyst [4]. The decision whether to use an anterior-only, posterior-only or combined approach is determined by the degree of proximal extension of the cyst (if higher than the third sacral body, a combined anterior–posterior or anterior-only approach should be used), whether or not the cyst had been infected and was adherent to surrounding structures (bladder, ureters, rectum), and whether or not the cyst underwent malignant transformation [4]. A combined approach is specifically advised when the tailgut cyst is large, as occurred with our first patient. Using this method is rare, because cysts are usually smaller (mean of 4.4–5.5), operation time is longer, and it requires the shifting of the patient’s position during the procedure [4,17]. Performing a coccygectomy has its proponents and adversaries. Some authors maintain that it improves surgical exposure and the decreases risk of recurrence due to the coccyx harboring a nidus of totipotential cellular remnants that may evolve into a recurrent cyst, but most surgeons elect to preserve it unless en bloc resection is required for malignancy or the cyst is densely adherent to it. Coccygectomy may be also required if the tumor is large (greater than 10 cm) or is difficult to access. In our patients’ cases, both of them had their coccyx bone excised.

During a dissection, the perforation of the tailgut cyst is possible, especially if the tumor infiltrates surrounding tissues. It was reported by Shah et al., as was observed in our case, that an odorless, thick fluid resulted from perforation [18]. If this occurs, taking a sample for culture is mandatory.

Postoperative complications sometimes occur, especially while using a posterior approach, including wound seromas, infections, or lower limb weaknesses [17]. In our patients’ cases, no postoperative complications were reported; however, in the first patient, lower limb ischemia was suspected, but it was ruled out after using a Doppler ultrasound.

## 4. Conclusions

In conclusion, tailgut cysts are a very rare condition, and due to nonspecific symptoms and rare incidence there is a possibility of diagnosis delay. Risk of malignancy is higher than has been estimated; therefore, increasing knowledge about these lesions is necessary to prevent physicians from overlooking them. We presented two cases of tailgut cysts, with the first being a large tumor excised using a complex procedure of a combined approach, and the second coexisting with endometriosis, which is to our best knowledge the first described case of these two diseases coinciding. The variety of cases, description of different symptoms and surgical approaches make our study an important source of knowledge for diagnosing and treating these very rare tumors.

## Data Availability

The original contributions presented in this study are included in the article, further inquiries can be directed to the corresponding author.
